# Lebanese Population Exposure to Trace Elements via White Bread Consumption

**DOI:** 10.3390/foods8110574

**Published:** 2019-11-14

**Authors:** Nada Lebbos, Claude Daou, Rosette Ouaini, Hanna Chebib, Michel Afram, Pierre Curmi, Laurence Dujourdy, Elias Bou-Maroun, Marie-Christine Chagnon

**Affiliations:** 1 Lebanese Agricultural Research Institute LARI, Heavy metals Department, Beirut PO Box 90-1965, Lebanon; lebbosnada@hotmail.com (N.L.); lari@lari.gov.lb (M.A.); 2 Lebanese University, Faculty of Science II, Laboratoire d’Analyse Chimique (LAC), Fanar PO Box 90656, Lebanon; claudedaou@hotmail.com (C.D.); rouaini@ul.edu.lb (R.O.); hchebib@hotmail.com (H.C.); 3 AgroSup Dijon, Univ. Bourgogne Franche-Comté, Biogéosciences UMR 6282, F-21000 Dijon, France; pierre.curmi@agrosupdijon.fr; 4 Service d’Appui à la recherche, AgroSup Dijon, F-21000 Dijon, France; laurence.dujourdy@agrosupdijon.fr; 5 AgroSup Dijon, Univ. Bourgogne Franche-Comté, PAM UMR A 02.102, Procédés Alimentaires et Microbiologiques, F-21000 Dijon, France; 6 Univ. Bourgogne Franche-Comté, INSERM U1231, NUTOX, Derttech “Packtox”, AgroSup Dijon, F-21000 Dijon, France; marie-christine.chagnon@u-bourgogne.fr

**Keywords:** survey, trace elements analysis, Lebanese pita, human exposure, risk characterization

## Abstract

The objective of this study was to assess Lebanese population exposure to trace elements (TEs) via white pita consumption. A survey of white pita consumption was achieved among one thousand Lebanese individuals, grouped into adults (above 15 years old, men, and women) and young people (6–9 and 10–14 years old). The most consumed pita brands, labeled B1, B2, and B3, were selected. Levels of TEs (i.e., As, Cd, Co, Cr, Hg, Ni, and Pb) in B1, B2, B3 pitas were measured. The highest contents of TEs in pitas were: Ni (1292 µg/kg) and Co (91 µg/kg) in B1; As (400 µg/kg) and Cd (< 15 µg/kg) in B2; Cr (363 µg/kg), Pb (260 µg/kg), and Hg (0.89 µg/kg) in B3. The pita brand B3 was the source of the highest TEs exposure, except for Ni for which it was B1. Daily exposures to TEs due to the fact of pita consumption were compared to safety levels. There were no safety concerns for Hg, Cd, Cr or Co (except the 95th percentile of 6–9 years old). An excess of the Ni tolerable daily intake was observed for the most exposed populations. The very low margins of exposure for As and Pb suggest a worrying risk for the Lebanese population.

## 1. Introduction

Bread is a source of energy and minerals, some of them being essential for the body. Essential minerals found in Lebanese bread are Cu, Fe, Mn, Zn, and Se which account for 36%, 25%, 30%, 22%, and 32% of respective recommended dietary allowance (RDA) values given for these elements [1].

Besides the quality of bread as nutriment, its safety must be assured and monitored in terms of putative contaminants like trace elements (TEs).

Indeed, trace elements represent the main food chemical contamination and are globally recognized as a public health hazard [2]. They occur either from natural process (lithogenic and pedogenic routes) and/or anthropogenic activities. The latter include industrial, agricultural (pesticides, fertilizers), and untreated wastewater [3]. This leads to the contamination of the different environmental matrices, including water, soil, air, and plants [4,5]. In general, plants can uptake TEs mainly by absorbing them from polluted soils, and transporting them further to its different parts (roots, stem, leaves, and grains) [6]. The bioaccumulation of TEs in plants, especially those that enter the food chain, can be dangerous to human health due to the fact of their eventual toxicity [5].

Traces elements such as lead, arsenic, cadmium, and mercury are relevant toxic elements [7]. According to the existing literature, it is mandatory to quantify the contents of trace elements in foodstuffs in order to estimate dietary exposure, an essential step for risk assessment related to food consumption [2,8].

Currently, many studies have been undertaken in different countries to assess exposure to TEs occurring in their relative foodstuffs such as France [9], Spain [2], Italy [10], China [11], Saudi Arabia [12], and Lebanon [13]. In addition, the European Food Safety Authority (EFSA) panel on contaminants in the food chain has given its scientific opinion on the risks to public health related to the presence of Ni, Hg, and As in food [14,15,16]. Such studies should be representative of specific food as consumed by the population. In France, total diet studies (TDS) are national surveys routinely realized to assess public health risks associated with substances such as contaminants in food. They underline the necessity to reduce exposure to elements such as Cd, Al, Ni, As, and Co by a diversified diet (food items and origins) [17].

Nasreddine et al. [13] studied, using the TDS approach, the dietary exposure of the adult Lebanese urban population to six essential micro-nutriments (i.e., Co, Cu, Fe, Mn, Ni, and Zn) and two toxic heavy metals (i.e., Cd and Pb). This study constitutes a first estimate of the consumer exposure to trace elements through the diet in Lebanon. These authors determined that the average urban adult daily bread consumption was 136.8 g/day and that mean exposure was found to be satisfactory for most trace elements. However, the survey included only adult people living in the district of Beirut, thus preventing the extrapolation of the results to the whole country; indeed, agricultural areas are sections of high bread consumption.

Previous studies in Egypt, Iran, South Africa, Nigeria, and Portugal determined TEs contents in bread [18,19,20,21,22]. In Lebanon, Bou Khouzam and co-authors [1] determined the contents of 20 elements in three varieties of Lebanese bread (i.e., white, brown pita, and Saj bread) throughout the country during the wet and dry seasons. Toxic elements such as Cd and Pb were below maximum levels set by the Lebanese regulatory agency, (The Lebanese Standards Institution—Libnor) [23].

Bread is an integral part of the daily diet, but the types of bread and amounts ingested vary according to the age, sex, and socio-economic status of the individual. As a result, and as consumer behavior evolves over time, there is a need for up-to-date information on daily ETs intake via Lebanese bread consumption throughout Lebanon, including rural areas.

The Lebanese standards [23] provide only Pb, Cu, and Cd maximum levels in bread as food item contamination vectors. However, other TEs could be present [1,13]. As exposition represents the combination of consumption of bread potentially contaminated, it is of first interest to compare exposure with safety levels in order to monitor any safety preoccupation concerns for the Lebanese population. For this reason, this study was directed by a public institute (Lebanese Agricultural Research Institute—LARI) to assess exposure to trace elements via Lebanese pita consumption.

As, Cd, Co, Cr, Hg, Ni, and Pb were selected in this study based on previous studies of trace element composition of Lebanese bread [1,13] and based on the Lebanese bread standard Libnor, NL 240 [23]. In contrast to heavy metals known to be toxic, such as Pb, Cd or Hg, some elements, such as Cr and Co, are essential elements giving benefit to the body, the liver being a valuable source of elements [24]. Co is a transition metal with two oxidation states Co (II) and Co (III) and is essential as part of vitamin B12 (cobalamin) involved in folate and fatty acid and the metabolism of proteins and nucleic acids [25]. In regards to Cr, the Cr (III) form has been postulated to be necessary for the efficacy of the insulin-regulating metabolism of carbohydrates, lipids, and proteins [26]. However, EFSA [27] in 2014 concluded there was no evidence of a beneficial effect associated with Cr intake in healthy, normoglycemic subjects.

Two risks may occur with trace elements: one is insufficient intake and one is excess intake. In this study, we focused on risk characterization due to the fact of excess intake via pita consumption.

Therefore, the objective of this study was to assess exposure to the selected trace elements (TEs) of the different Lebanese population categories via pita consumption. First, a country survey of pita consumption was achieved among one thousand individuals, grouped into adults (above 15 years old, men and women) and young people (6–9 and 10–14 years old). Second, levels of the seven selected trace elements in pitas from the three most consumed brands were measured. Third, using the survey data, population categories of exposures to TEs via white pita consumption were calculated and compared to the international health-based guidance values: tolerable daily intake (TDI), tolerable weekly intake (TWI) or toxicological reference points, such as bench mark dose limit (BMDL), to evaluate safety concerns.

## 2. Materials and Methods 

### 2.1. Survey

**Figure 1:**
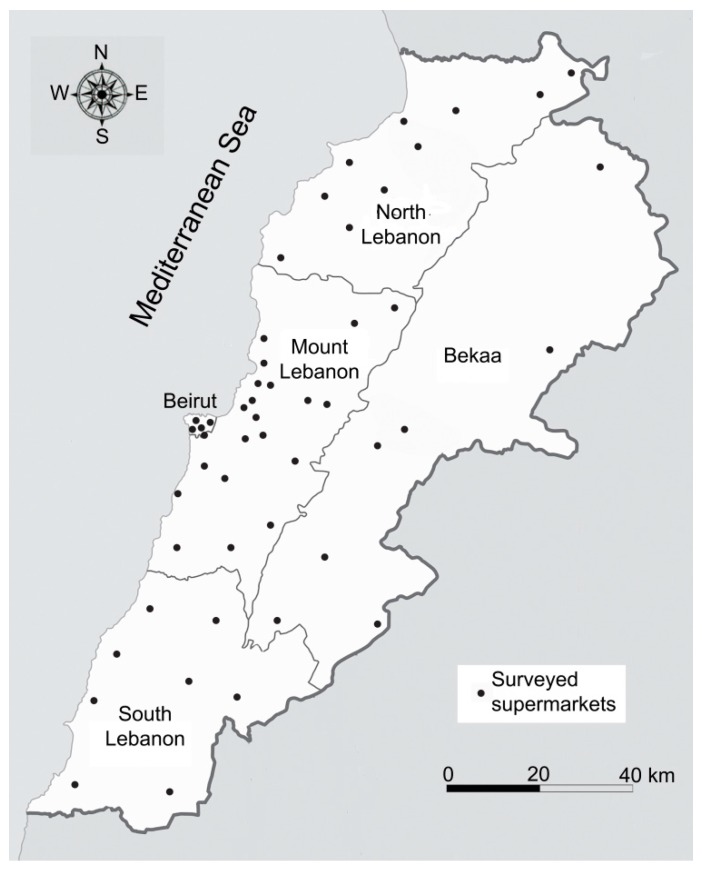
Location of the fifty surveyed supermarkets in Lebanon within the five administrative regions according to population density.

A questionnaire survey about the daily consumption of pita bread was conducted on a target sample of 1000 people. They were selected randomly in 50 supermarkets over the five administrative regions of Lebanon, taking into consideration the population distribution [28]: 41% in Mount Lebanon, 20% in North Lebanon including Akkar, 17% in South Lebanon including Nabatieh, 13% in the Bekaa, and 9% in Beirut (Table 1 and Figure 1). The individuals were represented by 50% men, 50% women, and grouped as follows: 800 adults (> 15 years old) and 200 young people (between 6 and 15 years old).

Data were collected over a two-month period, from 14 April 2017 to 5 June 2017. The team of interviewers was composed of 8 data collectors trained prior to the fieldwork by a supervisor. Participants were eligible for the study if they were Lebanese, had been living in Lebanon for at least fifteen years before the date of the survey, and did not have a chronic or serious illness.

In each supermarket, eligible participants were interviewed face-to-face by a data collector who instantly filled an offline questionnaire on an electronic tablet, using multiple-choice questions and figures (e.g., size of the pita). In the case of children, parents aided us in collecting survey data.

In addition to information on the daily consumption of pita bread, age, gender, body weight, and socio-economic status of the interviewed individuals, data were also collected on the level of education, address (urban or rural), job description, and health status (e.g., smoking habit, alcohol consumption and physical activity of the individual). Each questionnaire consisted of 44 questions (Supplementary Materials, Survey) which required approximately 7 min to complete. The collected answers were transmitted in the “iSurvey” application for data harmonization.

### 2.2. Chemicals

Standard solutions (1 g/L) of Hg, As, Co, Cr, Cd, Ni, and Pb were purchased from Fluka. In addition, HNO_3_ 65%, H_2_O_2_ 30%, and the BCR (Community Bureau of Reference) certified reference material for bread trace elements (BCR-191) were purchased from Sigma–Aldrich. Deionized water, obtained from a BOECOpure UV/UF water purification system, was used in all experiments.

### 2.3. Sampling for Trace Elements Analysis

Based on the survey data, three Lebanese white pita brands were determined as the most consumed by the Lebanese population. These brands were tagged as B1, B2, and B3.

Three randomly sampled bags of each of the selected pita brands were collected. Each bread bag had seven pitas where three were randomly picked. Each pita was cut into 12 circular sectors with a plastic knife on a polyethylene plate to prevent trace element contamination. Two pieces were randomly drawn for analysis, creating 18 (3 bags × 3 pitas × 2 pieces) circular pita pieces for each brand. Before sample digestion, pita pieces were ground into powder using an agate mortar.

### 2.4. Sample Digestion for Trace Elements Analysis

Digestion of samples was done using an Anton Paar microwave (multiwave 3000—Rotor 8SXF 100, Graz, Austria). Samples of 0.5 g each were directly weighed after grinding in 50 mL Teflon tube. Then, 7 mL of concentrated HNO_3_ and 1 mL of H_2_O_2_ were added to the preparation. The Teflon bombs were closed and placed in the digester at 200 °C for 15 min (1000 W). Once the Teflon tubes had cooled for at least 45 min, 3 mL of the digested samples were transferred to 25 mL polyethylene tubes and diluted with HNO_3_ 1% before the trace elements analysis. Each sample was digested in triplicate.

### 2.5. Analysis of Trace Elements 

The determination of trace elements (As, Co, Cd, Cr, Pb, and Ni) in pita samples was performed using an Atomic Absorption Spectrometer (AAS, Stafford House, UK) Zeeman Graphite Furnace GF95Z Thermo Electron corporation M series. Each pita sample was digested in triplicate and each digested sample was analyzed in triplicate. Mercury was analyzed by direct mercury analyzer DMA 80 Milestone (Sorisole, Italy) using 0.2 g of pita sample.

To guarantee the method reliability, a certified reference material (CRM; BCR-191) underwent the same digestion protocol and was analyzed at the same time as the pita samples. The recovery percentages obtained for the reference material were 93.2% for Hg, 110.8% for Pb, 86.3% for Ni, and 110.1% for Cd. For the other elements, As, Co, and Cr, for which reference materials were not available, bread samples were spiked by a known concentration (0.1 mg/L) of each of the above listed elements before digestion. The spiked bread was treated and digested in the same way as the bread samples and analyzed using an AAS. The recovery percentages were 103.08% for As, 101.29% for Co, and 96.37% for Cr.

### 2.6. Exposure Determination

An exposure calculation was conducted for each respondent. They took into account the quantity of bread consumed by person, body weight, and the metal content of the consumed bread according to its brand. Results were expressed in terms of the median and 95th percentile using Ms-Excel for different population categories.

### 2.7. Statistical Analysis

In order to avoid a lack of representativeness of the selected samples of the surveyed population, a weighting adjustment technique was applied using auxiliary variables such as gender, age, and region by comparing the observed frequency distribution of each variable with its population distribution [29].

Data processing and statistical adjustments were analyzed using Sphinx IQ2 (Le Sphinx®, Chavanod, France) for survey data and R Studio version 1.0.153 with R version 3.5.0 [30,31].

Kruskal–Wallis non-parametric tests [32] completed by a multi-comparison Fisher’s test (α = 0.05) were conducted to test for significant differences in consumption by region. Analysis of variances (ANOVAs) completed by a multi-comparison Tukey’s test (α = 0.05) were also executed to test differences in trace element contents among brands. They were performed with R using the “agricolae” package [33].

Median and percentile non-parametric tests (α = 0.05) [32] were realized to check significant differences in the level of trace element exposures between age and gender. They were performed using R and the “rcompanion” package [34].

Principal component analysis (PCA) was applied to examine the relationships among the trace element contents of the breads using XLSTAT 2018. The results are presented by loading and score plots.

Multivariate correspondence analysis was done to explore relationships among consumption behaviors and socioeconomic data [35].

## 3. Results

### 3.1. Survey Results

Based on a target sample of 1000 people, the surveyed sample consisted of 992 individuals with the following demographic characteristics: a sex ratio of 0.992, a proportion of 19.4% young people (8.9% children aged from 6 to 9 years old and 10.5% teenagers aged from 10 to 14 years old) and 80.6% adults, and 70.6% living in an urban area. Compared to the overall Lebanese population (where the sex ratio is 0.962, 23% are young people, 77% adults, and 87% live in urban areas [36]) there was a significant difference risk of 5% in terms of young people/adults and rural/urban ratios between the surveyed sample and the population distribution of Lebanon, which could lead to a bias. Consequently, a weighing adjustment in respect to these variables was performed to make a possible inference at the population level. The weighted numbers of respondents corresponded to 228 young people (i.e., 95 children and 133 teenagers) and 764 adults.

White pita was the type of bread most consumed according to the surveyed sample: 77% reported white pita consumption and 23% brown or other. We limited our study to the 762 individuals consuming mainly white pita (W). This population also consumed brown pita (B) and other types of pita (O) in smaller proportions. The relative proportions of these different consumption modes were as follows: W only > W + B > W + O > W + B + O with 88.8%, 8.9%, 1.8%, and 0.4%, respectively (Supplementary Materials, Table S1).

The survey makes it possible to estimate the consumption of white pita for the different categories of the population (Table 2). Adult men were the highest consumers followed by teenagers (10–14 years old) and women and children (6–9 years old): daily white pita consumption medians were 282, 143, 126, and 71 g/day, respectively. The 95th percentiles were 750 g/day for men and between 357 and 375 g/day for other categories. Maximum consumption was 1000 g/day for men and teenagers and 750 g/day for women and children. White pita brand consumption was significantly dependent on the age of the Lebanese population. Children consumed mostly the B2 or B3 brands, teenagers consumed mostly the B1 brands, and adults consumed mostly the B1 or B2 brands. The whole sample consumption by brand, including young people and adults, was B2 > B1 > B3 representing, respectively, 30.1%, 28.9%, and 17.1% of the total sample and the 46 other brands representing the remaining 24.9%.

**Figure 2:**
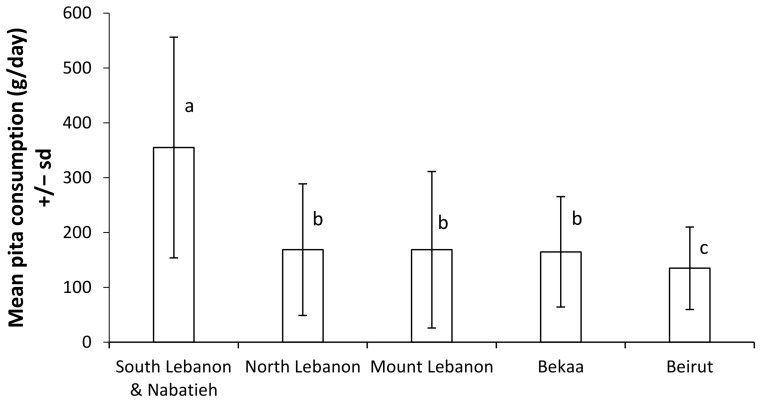
Lebanese white pita consumption according to region. Letters represent homogeneous groups determined by a Kruskal–Wallis test and post-hoc Fisher's test for least significant difference.

White pita consumption varied according to region (Figure 2), and the highest consumption corresponded to South Lebanon and Nabatieh (355 g/day). There were no significant differences between the North, Mount Lebanon, and Bekaa regions, which were two-fold lower than South Lebanon and Nabatieh. The lowest consumption was observed in Beirut (135 g/day). This last value is in agreement with the adult population of Beirut studied by Nasreddine et al. [13].

**Figure 3:**
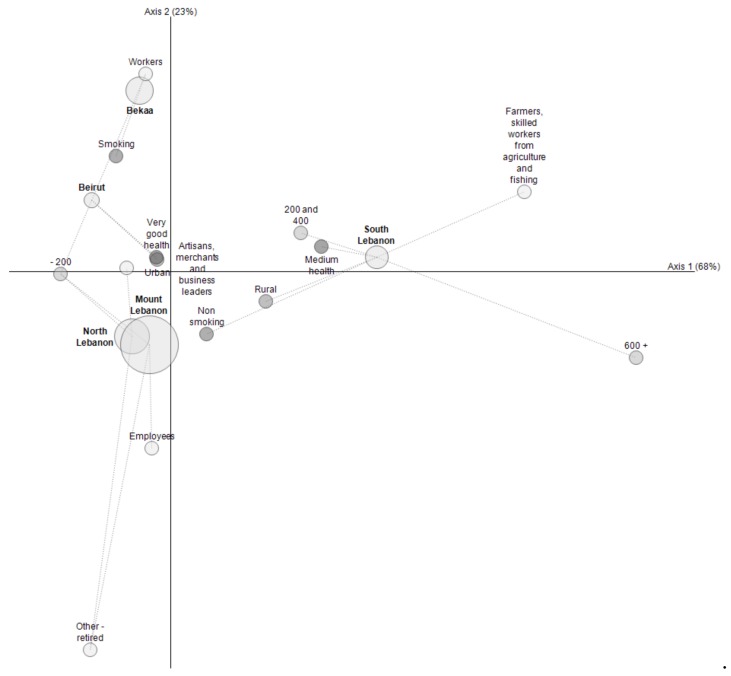
Results of crossings between regions, consumption, smoker or not, type of physical activity, socio-professional category, and area (urban or rural), using the most specific modalities as key views. Consumption is represented here by categories: less than 200 g/day (−200); from 200 to 400 g/day; from 400 to 600 g/day; and more than 600 g/day (600+). Sizes of circles are proportional to the number of observations by region.

Regardless of the region, large standard deviations were observed in the distribution of bread consumption; the reason could be that adults over 65 years old eat more bread, especially in rural areas (in South Lebanon, more than 600 g per day can be consumed, see Figure 3).

In order to better explore the relationships between adult consumption and regions, a data-mining tool was used, the purpose of which was to represent in only one table or figure the most significant results from several cross analyses (based on multivariate correspondence analysis [35]). A pivot variable was first selected—the region—then other variables were crossed with it. Figure 3 shows the results of crossing region and consumption, gender, socio-professional category, smoking habits, physical activity (4 categories: “No”, “Yes, strenuous”, “Yes, low”, and “Yes, medium”), and area (urban or rural), using the most specific modalities as key views.

In South Lebanon and Nabatieh, where consumption was the highest, it correlated to rural areas with more agriculture and elderly populations. The other parts were characterized by urban areas with high concentrations of workers and retired people.

In addition, 41% of total consumers of Lebanese white pita bread were smokers (at least one cigarette per day, with a distribution of 68% men and 32% women) and 35% were alcohol drinkers (at least one glass per day, with a distribution of 67% men and 33% women, data not shown). Those behaviors may affect health and the detoxification process of alcohol and smoking are indeed well-known inducers of several xenobiotic metabolism enzymes.

Among the adult population, 85% reported engaging in physical activity which was qualified as low to medium by women and medium to high by men (Supplementary Materials Figure S1).

The correlation between consumption and health was also evaluated (data not shown). The pivot variable was still pita consumption per day and the other variables were gender, region, professional activity (student, retired, etc.), and health (smoking, physical activity). 

From this study, two typologies of adult consumers can be drawn: i) Women, quite young (under 20 years old), non-smokers, and who felt very healthy according to the survey. They were living in urban areas, Lebanon North–Mount Lebanon, with some level of pre-secondary education. The consumption of those aged 15–64 years old per day was approximately 163 g of pita (corresponding to 1 or 2 pitas). ii) Men between 40 and 59 years old who felt healthy. They were living in rural zones, with a technical secondary level education. Their consumption was approximately 267 g of pita per day, corresponding to 3 to 6 pitas for those 15–64 years old.

### 3.2. Trace Element Contents in Pita

The levels of trace elements in the most consumed white Lebanese pitas (B1, B2, and B3) are shown in Table 3. B1 contained the highest level of Ni (1292.1 ± 0.2 µg/kg) and Co (91 ± 3 µg/kg). B2 contained the highest level of As (400 ± 7 µg/kg), while B3 contained the highest level of Hg (0.89 ± 0.06 µg/kg), Cr (363 ± 10 µg/kg), and Pb (260 ± 81 µg/kg). Levels of Cd were lower than the maximum levels defined by the Lebanese standard (200 µg/kg of bread). In contrast, Pb exceeded the maximum level (200 µg/kg) in B2 and particularly B3 (260 ± 81 µg/kg).

**Figure 4:**
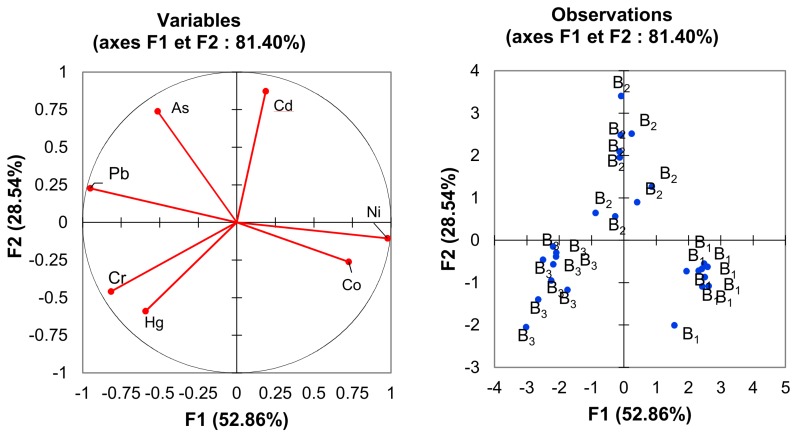
Principal component analysis showing trace element signatures of the B1, B2, and B3 pita brands.

In order to reveal the relationships among trace elements, a PCA was performed (Figure 4). 

Principal component analysis is a useful technique for exploratory data analysis, allowing to better visualize the variation present in a dataset with many interrelated variables. In our case, the dataset contained 27 observations (9 samples per brand) described by 7 variables (the trace elements content as mentioned in Table 3). Principal component analysis allows to see the overall “shape” of the data, identifying which samples are similar to one another and which are very different. It makes possible the identification of potential groups of samples that are similar and to work out which variables make one group different from another. When many variables correlate with one another, they will all contribute strongly to the same principal component [37].

Figure 4 shows typical plots from the PCA with a loading plot (on the left) and a score plot (on the right). The loading plot is used for interpreting relationships among variables and the score plot is used for interpreting relationships among observations.

All variables were well represented in the F1–F2 plot (sum of squared cosine above 0.75). The PCA shows that the following elements were correlated: Ni and Co, As and Pb as well as Cr and Hg. Lead and Co were anti-correlated, as well as Ni and Pb. There was no correlation between Cd and Co, Cd and Pb, Cr and As as well as Hg and As. The Pearson correlation matrix is provided in Supplementary Materials Table S2. We observed three separate groups: B1, B2, and B3. The dispersion in B2 and B3 was larger than in B1; this dispersion can be explained by a more homogeneous sampling for B1.

### 3.3. Trace Element Exposures

The population categories’ exposure to Cd, Hg, Cr, Co, Ni, Pb, and As via white pita consumption according the most consumed brands is presented in Supplementary Materials Table S3. Using statistics, we observed that, for most of the cases, the B3 brand was the origin of the highest trace elements exposure. In regards to nickel, B1 was the main source of the highest exposure. We further decided to keep B3 TE data to calculate exposures (Table 4) using a worst-case scenario for all element levels, except for nickel for which the B1 data were retained.

For young people, there was no significant difference among both populations in terms of exposure to any element. In contrast, we found significant differences between men and women for all elements except nickel. Men, at the 95th percentile, were always more exposed.

### 3.4. Risk Characterization (Intake Excess of TEs via Bread Consumption) 

In regards to essential elements with nutritional value, risk assessment can be calculated by insufficient intake or intake excess. This work focused only on intake excess using toxicological reference points or safety levels for each element case by case in order to determine if there is a safety concern due to the fact of an intake excess related to bread consumption in Lebanon. Of course, bread is not the only food consumed per day; however, in this study we show that the most exposed population—men and teenagers—can consume up to 1000 g of bread per day.

Considering chromium levels in bread, represented mostly by Cr (III)—the major form in food [38], exposures to Cr ranged from 1.21 to 9 μg/kg bw/day which represents only 3% of the European TDI [27] for the most exposed population (children at the 95th percentile) (Table 5) in regards to pita consumption. Concerning cobalt, only the exposure related to children lead to an excess (131%) of the French TDI (Table 6) [39].

Regarding cadmium, the most exposed population were children at the 95th percentile followed by teenagers and then men and women. The most exposed population represented 40% of the European tolerable weekly intake (TWI) fixed by the EFSA [40] (Table 5).

Exposures to mercury were also very low (ranging from 0.02 to 0.15 μg/kg bw/week) even for the most exposed population (children at the 95th percentile) leading to 11.5% of the TWI fixed by the EFSA [15].

However, before concluding on safety in this survey in regards to Hg, Cd, Cr or Co, it is important to take into account exposure from other food items in the context of the Lebanese population’s total diet.

Concerning nickel, the situation is already worrying when taking into account only pita consumption. Teenagers were the most exposed population followed by children and then adults, regardless of sex (Table 6). The European TDI was exceeded for all exposures for teenagers and men and only at the 95th percentile (3 to 4 fold) for children and women.

Table 7 shows that the margins of exposure (MOE) in regard to lead, determined with a BMDL_0.1_ based on neurotoxic effect [41], were very low (between 0.08 and 0.58) whatever the population. The most exposed populations in decreasing order were children, teenagers, men, and women. The calculated MOEs suggest a safety level of preoccupation regardless of the population in terms of lead exposure via bread consumption.

The same safety preoccupations can be observed with arsenic considering As is represented by its inorganic form. The EFSA has concluded that 0.3 to 8 μg/kg bw/day should be used as a point of reference [16]. Thus, these values were used for MOE calculation to assess risk characterization. Table 7 shows that the MOEs were very low regardless of the population in both scenarios. The most exposed populations were children followed by teenagers and then men and women. The MOE data suggest a worrying risk in regard to As exposure linked to pita consumption regardless of the population in this survey.

## 4. Discussion

### 4.1. Survey

The direct input of the answers to the questions asked by the data collectors on electronic tablets made it possible to calculate exposure values to TEs at the level of the individual insofar as we had all the necessary data at this scale: age, gender, body weight, daily intake, and brand of pita. The most recent works on this topic were based on a survey conducted in 2001 and for which the calculation of exposure from the intake was based on the average body weight of the whole surveyed sample [13,43].

This survey was based on a nationally representative survey, a real sample covering 992 Lebanese consumers. To our knowledge, this was the first survey assessing the pita diet in Lebanon in different Lebanese regions covering young people and adults. Previous studies focused on adults in the Beirut area only. There was a large standard deviation in the distribution of bread consumption among adults in all regions. This could be explained by the fact that people aged 65 and over eat a lot of bread.

The use of the questionnaire responses provided a good overview of pita consumption by region, age, and gender. It allowed for the drawing of typologies of people living in these regions too. The combination of estimates on consumption from the survey and the analytical results from pita experiments made it possible to do a risk evaluation.

The exposure study was done on a population consuming mainly white pita (762 individuals out of 992). The remaining population who did not consume white bread would be, in principle, more exposed to trace elements based on the article by Bou Khouzam et al. [1] which emphasizes that Lebanese brown bread is more contaminated than white.

### 4.2. Risk Characterization

In terms of risk characterization linked to the seven trace elements analyzed in the pita bread, a highly consumed foodstuff, two of the trace elements, Cd and Hg, appeared to not be a safety concern. The results for Cd are in accordance with the Nasreddine [13] study conducted on the total diet in an adult urban population. Bread may not be the main food involved in the highest exposure to Cd, as these authors showed that the main contributors to the dietary intake of Cd in a Beirut TDS were vegetables (46.8%) followed by breads and cereal-based products (30.9%). Same as for Cd, bread is not the main source of Hg, for which it is fish and seafood, which is a concern for high fish consumers [17] and, particularly, pregnant women [15]. We found between 0.70 and 0.89 µg/kg of Hg in Lebanese pita which is by far less than in the study of Mestek and co-workers [44] who found 13 µg/kg of Hg in bread in the Czech Republic.

In our study, total chromium was analyzed. The B3 pita contained the highest level of Cr (0.36 mg/kg of dry weight). It is known that the major form of Cr in food is Cr (III), and the major contributor of Cr is bread (8%) followed by bakery products. For children, it is milk (9%) followed by pasta (6%) [22]. In this study, the analytical data from the two brands (B1 and B2) were in accordance with Soares’ [22] team who quantified the total chromium contents (47.3 ± 20.0 μg/kg) of dry weight white bread samples.

Compared to Cr (VI), Cr (III) is considered an essential element for humans and authorized in food supplements in Europe without any upper limit [45]. But, in 2010, the EFSA [46] recommended that total Cr does not exceed 250 µg/day, a value established by the WHO [26]. Furthermore, Cr (III) can complex with lactate and picolinate, causing concern as it has been demonstrated to be genotoxic [27]. In contrast, Cr (VI) is known to be highly toxic and carcinogenic (Group 1, [47]). It also induces skin dermatitis, then the interconversion will be of relevance in terms of risk assessment.

Even if data on the presence of Cr (VI) in food are missing, it is considered that food is a large reducing medium and that oxidation of Cr (III) to Cr (VI) would not occur. Additionally, most of the Cr (VI) is considered to be reduced in the stomach to Cr (III) [27].

Humans are exposed to Cr mainly via food, but it can be present also in water. Concerning bottled water in Lebanese markets [48], a study of the physicochemical and heavy metal parameters in 32 samples of bottled waters, 25 of these samples showed values below 1 µg/L for Cr, while the other samples showed values ranging between 1 and 4 μg/L in the drinking water. Although naturally occurring Cr (VI) is rarely found in the environment, its presence in ground and surface waters, as a consequence of oxidation of Cr (III)-bearing minerals by Mn (IV) oxides, has been reported recently [49]. Furthermore, in terms of the total quantity found in bread, we do not know its respective part coming from the wheat flour or water used during the bread-making process. Wheat are among species that demonstrate the ability to bio-accumulate chromium the most, just after tomato [5]. An average value of Cr below 0.05 mg/kg of dry weight was measured in wheat seeds in Swedish long-term soil-fertility experiments between 1967 and 2003 [50].

The recipient might also be a source of contamination if Cr is released. Indeed, migration of chromium from steel material occurred in the past mostly from Asia (China) and was above the upper legal limits for food contact material [51].

The EFSA panel recommended the generation of data using sensitive analytical methodologies which specifically measure the content of Cr (III) and Cr (VI) in food and drinking water in different EU Member States [27]; the issue is the same for Lebanon. However, Cr speciation is difficult to conduct, especially in complex matrices. Chromium speciation depends on several factors such as pH, concentration, oxidation state, and complexation with natural components present in the matrix such as peptide [49]. Soares and co-workers [22] mentioned the presence of Cr (VI) in bread after extraction of Cr by alkaline solutions. However, Novotnik et al. [38] demonstrated that the only Cr species in bread is Cr (III) by spiking with enriched isotopes of ^53^Cr (III) and ^50^Cr (VI), a more accurate quantification, and using HPLC-ICP-MS. Thus, we made a characterization in regards to Cr (III) and, in this way, there were no safety concerns in this study for Cr (III) in Lebanese pita bread.

For cobalt, the exposure of young people under 15 years old at the 95th percentile exceeded the TDI by a percentage of 131%. Bread and cereals are significant source of Co exposure (18.1%), with a daily intake of cobalt estimated to be around 11.4 µg/day according to the Nasreddine study [13]. Indeed, in their study they found 6.36 µg/kg of cobalt in bread and cereals. In this study, the level of Co was 91 µg/kg for the B1 pita brand, which is 7 fold more than in Australian bread and 13.5 fold higher than the data from the Nasreddine study [13] but 1.8 fold less than in Ethiopian bread [52].

In Lebanon, imported flour samples used in Lebanese white pita presented values for cobalt between 2 ± 2 and 33 ± 7 µg/kg which is far less than in the pita. Cobalt contamination can also occur during bread preparation via the water used. The mean level of Co in tap water is approximately 9 µg/L (IRAL, personal communication). In 2013, the Environmental Working Group’s (EWG) mission detected Co in only one sample (6 analyzed) and found 1.01 µg/L [53]. Furthermore, cobalt can also be released from food contact materials (FCMs) as it is used in ceramics [54]; thus, we can wonder if cobalt migration can occur from recipients used for making bread in Lebanon.

Several forms of cobalt (II) (sulphate, dichloride) are classified as presumed human carcinogens via the inhalatory route [55]. The IARC classified the soluble form of cobalt II in class 2B but as metal, Co was classified in 2A [56]. Although Co (II) salts are able to induce genotoxicity in vitro and in vivo after oral or parenteral exposure; however, there is a lack of carcinogenesis studies in humans and animals following an oral route [57]. The EFSA concluded that it cannot be excluded that cobalt could have a non-threshold toxicological effect [58]. In France, ANSES made the recommendation to reduce food exposure to cobalt in infants under 3 years old [39]. Thus, for cobalt, the daily intake from all dietary sources may be quite pertinent to monitor in order to determine the total exposure of young people under 15 years old in Lebanon.

In regards to nickel, the B1 brand contained the highest value (1292 µg/kg), which was above values found in studies for Nigerian, Ethiopian, and Spanish bread [52,59,60] but under those obtained in Iranian bread [61]. However, for another brand the value (365 µg/kg) was in the same range.

The average daily intake of nickel was estimated at 126.27 µg/day bread and cereals contributing to a 54.73 µg/day Ni intake in the Lebanese adult urban population [13].

In a French TDS study regarding the infant population, the value determined in bread was approximately 69.5 µg/kg [39]. The contribution of drinking water to total exposure to nickel was very small across dietary surveys and age classes [14]. We do not have an explanation, but this high level could be due to the recipient used by bakers. Using the TDI 2.8 µg/kg bw/day [14] for oral chronic exposure, the data demonstrate that the percentage of Ni TDI was exceeded 3 to 4 fold for all populations with the 10–14 years old range being the most exposed. These data are in accordance with the TDS studies also showing a safety concern in France for very young people (<3 years old) [39].

Nickel compounds and metallic nickel are classified as human carcinogens, respectively, group 1 and group 2B after inhalation [62]. Available studies in animals suggest that exposure to nickel salts lead to renal effects and increases neonatal mortality with the kidney being the principal target. Reproductive and developmental toxicity were the critical effects after chronic oral exposure retained for risk characterization. However, oral exposure to nickel salts could be at the origin of contact dermatitis for sensitive populations. Indeed, oral nickel can also cause an oral and chronic effect in people dermally sensitized [14].

In humans, absorption of Ni varies according to the method of exposure (in water it is 40 fold more absorbed compared to food; 0.7% and the bioavailability varies with the food type [63]). There are no maximum levels in food for nickel; in contrast, a value of 20 μg/L is fixed in water intended for human consumption and in mineral waters in Europe [64,65].

In this study, arsenic exposure was more worrying, as we found a high level of arsenic for all brand analyzed (235–400 µg/kg) which is around 63 fold higher than those found in a bread study in the UK [66] and 32 fold higher than those mentioned in a French infant TDS study [39]. Bou Khouzam et al. [1] mentioned seasonal variation in the arsenic concentration in bread (33 µg/kg in dry season, which is 4 fold higher than in wet season). Arsenic was not studied by Nasreddine et al. [13] and calculated MOEs were very low for all bakeries and the population. These data are in accordance with the data and risk characterization in 19 countries in Europe [16], for example, in France where there is a safety concern with MOEs lower than 36 [39], suggesting that the situation in Lebanon is worrying.

The inorganic form of As, which is known to be toxic, may cause human malignancies. It was classified as carcinogenic (group 1) in Reference [67]. The toxicity, due to the inorganic form, occurs by an epigenetic mechanism, and there is increasing evidence that early life exposure affecting fetal and infant growth [68] may cause chronic disease later in life [69]. This highlights the urgent need to obtain data on the effect of the toxicity of As on development [39]. Arsenic passes through the placental barrier in humans [70], resulting in similar levels in both the fetus and mother. Both the inorganic form and its methylated metabolites are transferred [71]. The effect of As on the endocrine system has also been investigated for many years showing a disruption on the gonadal, adrenal, and thyroid endocrine systems [72]. Furthermore, it is has been shown a toxic effect due to an interaction between Cd and As [73,74]. Drinking water, crops irrigated with contaminated water, and food prepared with contaminated water are sources of exposure [75].

The EFSA’s recommendation is to reduce inorganic arsenic exposure in food and develop robust validated methods to determine the inorganic form [16]. We do not distinguish between organic and inorganic form of As in this study. It is known that arsenic intake is higher from solid foods than from liquids.

Furthermore, speciation plays a major role in determining the amount of arsenic absorbed after consumption of As contaminated food as well as bioavailability with significant interspecies differences for organic arsenic. Regards to inorganic form, absorption can be high as demonstrated with contaminated rice [76,77].

Representative data on speciation are indeed scarce. The EFSA panel assumed a proportion of the contaminant to inorganic form (the toxic one) to vary between 50 (best case) to 100% (worst case) of the total arsenic reported in food commodities in Europe, other than fish and seafood [16]. Furthermore, in bread, 92% of the inorganic form has been found [78]. Cereals and cereal-based products were identified as largely contributing to the daily exposure to inorganic arsenic, as it was quantified as 53 µg up to 72 µg of arsenic per kilo for all cereals and cereal product categories. In wheat samples, it was demonstrated that 91% to 95% of As was in the inorganic form [77,78]. In wheat plants cultivated in the Bekaa region, the element As presented values between 288.3 ± 14.7 and 743.7 ± 25.4 mg/kg. In the irrigation water used in the Bekaa sites cultivated for wheat plants, the As content varied between 0.8 ± 0.3 μg/L and 1.2 ± 0.2 μg/L (IRAL data). Indeed, the level of As in drinking water in Lebanon varied between 1.2 and 4.5 µg/L [79]. Water used for making bread can be the main contributor to the level of arsenic in the pita with the main form recovered in drinking water as inorganic. It is important to note than that change in the speciation of As can occur during food preparation [80].

Concerning the level of lead in the different brands: B2 and B3 contents were more than 2.5 fold higher than B1 (74, 203, and 260 µg/kg, respectively). The order of magnitude of B1 is in accordance with Turkish, Iranian, Ethiopian, and Spanish studies on bread [52,59] and 7 fold more higher than in data on the Czech Republic [44]. The B3 pita lead content was 32 fold higher than Lebanese data [13] which focused on Beirut. The authors noticed that the lead exposure in Beirut was even two fold lower compared to a previous study [43]. Thus, our data were not in accordance, but it took into account all the Lebanese regions including rural ones. The lead origin may be from wheat. Several samples of Lebanese wheat cultivated in the Bekaa region were studied, showing values of lead varying between 4.5 and 23 µg/kg but far less than in the bread, noting that the lead values in Lebanese wheat samples were lower than the values showing in wheat samples in Saudi Arabian markets (2.81 ± 0.08 mg/kg dry weight) [12]. While, the lead levels, measured in five wheat flour samples in Lebanese markets varied between 0.0045 ± 0.002 and 0.023 ± 0.016 mg/kg dry weight, they were lower than values presented in wheat flour samples in the Canary Islands and used for bread production (varying between 0.037 ± 0.013 and 0.056 ± 0.045 mg/kg fresh weight) [81].

Furthermore, lead can also be a contaminant issued from recipient food contact materials or water used for bread making.

Human data show that the developing nervous system is a critical target in children. Various water soluble and insoluble lead can induce tumors in rodents, especially renal tumors as a carcinogenic/promoter; brain gliomas were also observed. Classified lead compounds are probably carcinogenic to humans on the basis of limited evidence of carcinogenicity in humans and sufficient evidence in animals [56].

It is important to note that due to the long lead half-life, chronic exposure is of most concern and children absorb lead after ingestion to a much greater extent (70% of the amount of lead ingested) than adults (20%) [82].

All MOEs calculated with exposure only via pita consumption in this study were very low, but in the same range than in France and Europe [17,39,41], suggesting an exposure safety concern and that it is necessary to decrease Pb exposure, for example, by varying the diet due to the difficulty in reducing Pb contamination.

For both lead and arsenic, this contamination occurred for all bread brands analyzed in this study and there is a safety concern (Supplementary Materials Table S3).

## 5. Conclusions

This was the first study conducted all over Lebanon showing the exposure of different categories of Lebanese population to trace elements via bread. A survey of daily consumption of pita bread was conducted on a sample of 992 people. Among the four categories (i.e., children, teenagers, women, and men), white bread was the most consumed. Men consumed the highest quantity (282 g/day). The survey revealed that three brands of white pita were the most consumed: B1, B2, and B3.

Seven trace elements (i.e., As, Cd, Co, Cr, Hg, Ni, and Pb) were quantified in pitas coming from B1, B2, and B3. B1 was characterized by high levels of Ni and Co. B2 was characterized by the presence of As and Cd, and B3 was characterized by the presence of Cr, Hg, and Pb. Lead concentrations exceeded the authorized limits of the Lebanese standard for bread in the two brands. Further analysis should be performed on soil, wheat, flour, and water in order to determine the source of the contamination and to reduce exposure.

The B3 brand was at the origin of the highest trace element exposure, except for nickel for which B1 was retained. In terms of risk characterization, there was no safety concern due to the fact of pita consumption for all studied categories with Hg, Cd, Cr, and for Co except the 95th percentile of the children category (6–9 years old). Nickel exposure showed an excess of the TDI up to 4 fold for the most exposed populations (95th percentile). There were safety concerns related to As and Pb, as the margins of exposure were vey low. To our knowledge, this is the first time that exposure to As was studied in the Lebanese population. To confirm these observed data, it is urgent to analyze the inorganic arsenic form levels in bread. Experiments are currently being run to study any cocktail effects (synergistic) using pertinent bioassays due to the exposure to the sum of trace element identified in bread. The ultimate objectives are to protect the Lebanese population by reducing exposures to populations particularly exposed to TE presenting safety concern and to make recommendations, as bread is only one part of the daily diet.

## Figures and Tables

**Table 1:** Sampling strategy according to the population distribution.

Regions	Population Distribution *		Estimated Sample		Surveyed Supermarkets	Realized Questionnaires	
	*n*	%	*n*	%	*n*	*n*	%
Beirut	361,366	9.6	90	9.0	5	82	8.3
Mount Lebanon	1,484,474	39.5	410	41.0	20	410	41.3
North Lebanon	763,712	20.3	200	20.0	10	200	20.2
South Lebanon	659,718	17.6	170	17.0	8	170	17.1
Bekaa	489,865	13.0	130	13.0	7	130	13.1
Total	3,759,136	100	1000	100	50	992	100

* Lebanese Population, Central Administration for Statistics (CAS) Living Conditions Survey 2007 [28].

**Table 2:** Daily white pita consumption according to population categories and repartition by brands.

Population Categories	Individuals		Daily White Pita Consumption					Brands Repartition				
	*n*		g/Day					%				
		min	M	95th P	max	m	CI 95%	B1	B2	B3	Others *	Total
Children	91	63	71	357	750	113	94-131	24	31	30	14	99
Teenagers	113	31	143	375	1000	171	147-196	40	29	12	19	100
Women	258	31	126	375	750	167	153-181	29	31	16	24	100
Men	300	31	282	750	1000	282	260-304	29	30	13	27	99
Young people & adults	762	31	143	500	1000	206	195-218	28.9	30.1	17.1	24.9	101

M = median; 95th P = 95th percentile; min = minimum; max = maximum; m = mean; CI 95% = 95% confidence interval. * ”Others” correspond to an array of the 46 other pita brands.

**Table 3:** Trace element contents in white pita bread for the three major brands on the Lebanese market.

Trace Elements	Major Pita Brands			Limit of Detection (µg/kg)
	B1	B2	B3	
		(µg/kg Dry Weight)		
Cd	<LOQ	<LOQ	<LOQ	5
Hg	0.7 ± 0.1 ^b^	0.70 ± 0.15 ^b^	0.89 ± 0.06 ^a^	0.04
Cr	<LOQ	<LOQ	363 ± 10	16
Co	91 ± 3 ^a^	87 ± 5 ^a^	84 ± 2 ^a^	5
Ni	1292.1 ± 0.2 ^a^	364.82 ± 0.02 ^b ^	<LOQ	16
Pb	74 ± 5 ^c^	203 ± 30 ^b^	260 ± 81 ^a^	21
As	235 ± 22 ^c^	400 ± 7 ^a^	321 ± 20 ^b^	16

LOD = Limit of detection. Limit of quantification: LOQ = 3 × LOD. Values are the mean ± SD (*n* = 9/group) trace elements level. Statistical comparisons were done by Kruskal–Wallis with Tukey’s post-hoc analysis. Significantly different (*p* < 0.05) values among brands per element are indicated by different letters.

**Table 4:** Population categories’ exposure to trace elements via white pita consumption: Cd and Hg are expressed in µg/kg body weight/week while Cr, Co, Ni, Pb, and As are expressed in µg/kg body weight/day

	Population Categories’ Exposure to TEs via White Pita Consumption							
TEs	Children		Teenagers		Women		Men	
	M ^1^	95th P ^1^	M ^1^	95th P ^1^	M ^1^	95th P ^1^	M ^1^	95th P ^1^
**Cd**	0.19	1.00	0.20	0.56	0.13 ^a^	0.28 *	0.19 ^b^	0.47 **
**Hg**	0.03	0.15	0.03	0.09	0.02 ^a^	0.04 *	0.03 ^b^	0.07 **
**Cr**	1.74	9.00	1.79	5.06	2.47 ^a^	2.47 *	1.72 ^b^	4.26 **
**Co**	0.41	2.09	0.42	1.18	0.28 ^a^	0.57 *	0.40 ^b^	0.99 **
**Ni**	2.80	10.26	4.06	12.26	2.77 ^a^	10.26 *	4.34 ^a^	12.26 *
**Pb**	0.69	3.59	0.71	2.01	0.48 ^a^	1.77 *	1.23 ^b^	3.04 **
**As**	1.54	7.97	1.58	4.49	1.07 ^a ^	2.19 *	1.53 ^b^	3.77 **

The B3 brand, the origin of the highest trace elements exposure, was chosen for comparison with toxicological reference values except for nickel for which the B1 brand was retained. ^1^ M = median, 95th P = 95th percentile. Statistical tests were performed separately for young people and adults for the median and the 95th percentile. Values with different letters (a,b) in each row indicate a significant difference among medians at *p* < 0.05. Values with different stars (*,**) in each row show significant difference among 95th percentiles at *p* < 0.05.

**Table 5:** Population categories’ exposure to Cd and Hg trace elements via B3 white pita consumption and comparison with the tolerable weekly intake (TWI). Exposure values are expressed in µg/kg body weight/week.

Trace Elements	Population Categories’ Exposure to TEs via White Pita and Comparison with TWI															
	Children				Teenagers				Women				Men			
	M	M /TWI %	95thP	95thP /TWI %	M	M /TWI %	95thP	95thP /TWI %	M	M /TWI %	95thP	95thP /TWI %	M	M /TWI %	95thP	95thP /TWI %
Cd ^a^	0.19	7.6	1.0	40.0	0.20	8.0	0.56	22.4	0.13	5.2	0.28	11.2	0.19	7.6	0.47	18.8
Hg ^b^	0.03	2.3	0.15	11.5	0.03	2.4	0.09	6.7	0.02	1.5	0.04	3.1	0.03	2.3	0.07	5.6

The B3 brand, the origin of the highest trace elements exposure, was chosen for comparison with TWI values. M = median, 95thP = 95th percentile. ^a^ TWI for Cd = 2.5 µg/kg bw/week [40]. ^b^ TWI for methylmercury expressed as mercury, TWI for Hg = 1.3 µg/kg bw/week [15].

**Table 6:** Population categories exposure to Cr, Co, and Ni trace elements via white pita consumption and comparison with the tolerable daily intake (TDI). Exposure values are expressed in µg/kg body weight/day.

Trace Elements	Population Categories’ Exposure to TEs via White Pita and Comparison with TDI															
	Children				Teenagers				Women				Men			
	M	M /TWI %	95thP	95thP /TWI %	M	M /TWI %	95thP	95thP /TWI %	M	M /TWI %	95thP	95tthP /TWI %	M	M /TWI %	95thP	95thP /TWI %
**Cr ^a^**	1.74	0.58	9.0	3.0	1.79	0.6	5.06	1.69	1.21	0.40	2.47	0.82	1.72	0.57	4.26	1.42
**Co ^b^**	0.41	25.6	2.09	130.6	0.42	26.3	1.18	73.8	0.28	17.5	0.57	35.6	0.4	25.0	0.99	61.9
**Ni ^c^**	2.80	100	10.26	367	4.06	145	12.26	438	2.75	98	8.83	315	4.34	155	8.86	317

The B3 brand, the origin of the highest trace elements exposure, was chosen for comparison with TDI values, except for nickel for which the B1 brand was retained. M = median, 95thP = 95th percentile. ^a^ TDI for Cr (III) = 300 µg/kg bw/day [27]. ^b^ TDI for Co = 1.6 µg/kg bw/day [42]. ^c^ TDI for Ni = 2.8 µg/kg bw/day [14].

**Table 7:** Margin of exposure (MOE) to Pb and As trace elements via white pita consumption for the Lebanese population categories.

TEs	Population Categories’ Exposure to TEs via White Pita and Comparison with the Margins of Exposure																							
	Children						Teenagers						Women						Men					
	Median			95th Percentile			Median			95th Percentile			Median			95th Percentile			Median			95th Percentile		
	Value *	M0E 1	M0E 2	Value *	M0E 1	M0E 2	Value *	M0E 1	M0E 2	Value *	M0E 1	M0E 2	Value *	MOE 1	M0E 2	Value *	M0E 1	M0E 2	Value *	M0E 1	M0E 2	Value *	M0E 1	M0E 2
Pb ^a^	1.25	0.40	_	6.43	0.08	_	1.28	0.39	_	3.61	0.14	_	0.86	0.58	_	1.77	0.28	_	1.23	0.41	_	3.04	0.16	_
As ^b^	1.54	0.19	5.19	7.97	0.04	1.00	1.58	0.19	5.06	4.48	0.07	1.79	1.07	0.28	7.48	2.19	0.14	3.65	1.53	0.20	5.23	3.77	0.08	2.12

The B3 brand, the origin of the highest trace elements exposure, was chosen for the margin of exposure calculations. * Values of median and percentiles are expressed in µg/kg bw/day. ^a^ Pb margin of exposure calculated with BMDL_0.1_ = 0.5 µg/kg bw/day based on neurotoxic effect [41]. ^b^ As margin of exposure calculated with BMDL_0.1_ = 0.3 µg/kg bw/day for MOE 1 and BMDL_0.1_ = 8 µg/kg bw/day for MOE 2 [16].

## Supplementary Materials

The following are available online at https://www.mdpi.com/2304-8158/8/11/574/s1, Survey and questionnaire related to Lebanese bread consumption. Figure S1: Distribution of physical activity intensity by gender in adult population. Table S1-a: Cross-tabulation of consumed pita type occurrences for the total 992 respondents in the number of individuals. Table S1-b: Types of consumed bread for the subpopulation consuming mainly white pita (762 individuals). Table S2: Pearson correlation matrix of the principal component analysis. Table S3: Population categories’ exposure to Cd, Hg, Cr, Co, Ni, Pb, and As trace elements via white pita consumption according the most consumed brands.
