# Supplementary material for: Lebanese Population Exposure to Trace Elements via White Bread Consumption
Source: Foods. 2019 Nov 14;8(11):574. doi: 10.3390/foods8110574 (PMC6915579; doi:10.3390/foods8110574)
Supplement: Supplementary file 1 [file foods-08-00574-s001.pdf]

Manuscript Title: Lebanese population exposure to trace elements *via* white bread consumption
Supplementary data

Survey and questionnaire related to Lebanese bread consumption.
A study was conducted on the presence of metallic trace elements (ETM) throughout the Lebanese bread chain, from farm to fork. Some of these elements may cause health problems for the consumer, so the amount of Lebanese bread (likely to be contaminated) consumed per day and per person must be known, to assess the risk related to a route of oral exposure of trace elements.

Metallic trace elements are contaminants in the environment and foodstuffs, including bread. Once in excess of the environment, trace elements enter the soil, water or air and contaminate certain foods. They can cause a risk to human health if the consumer is too much exposed, for example, by ingesting cereals grown in contaminated soils. It is therefore important to monitor the presence of these contaminants in the bread and to know the exposure of the consumer by conducting a survey among the population in different regions of Lebanon.

Personal data collected will only be used for exposure calculations and will not be disclosed. Anonymity will be ensured throughout the study.

There is no intervention to undergo or therapeutic protocol to follow.

- **Do you have a chronic or serious illness?** □ Yes □ No
- **Do you eat Lebanese bread for more than 15 years?** □ Yes □ No
- **Do you live in Lebanon for more than 15 years? (Criteria for adults only)**

  □ Yes □ No

If you meet the above criteria, please complete this questionnaire, which will take you less than ten minutes

Date................................. investigator………………….

Part 1. Consumption of Lebanese bread

1. What is the kind of Lebanese bread eaten?

1 □ White bread 2 □ Brown bread 3 □ Other please specify: ....

2. What is the brand of bread most consumed?

1 □ Brand 1 2 □ Brand 2 3 □ Brand 3

4 □ Brand 4 5 □ Others please specify: ....

3. What is the size of pita consumed?

1 □ Small 2 □ Medium 3 □ Large

4. What is the number of pitas consumed per day?

1 □ 2 □ 3 □ 4 □ Others please specify: ....

Part 2. Sociodemographic data

5. Age: | __ | __ |

6. Gender: 1 □ Man 2 □ Woman

Foods **2019**, *8*, 574; doi:10.3390/foods8110574 www.mdpi.com/journal/foods

7. Place of residence:

1 □ Beirut 2 □ Mount Lebanon 3 □ North Lebanon

4 □ South Lebanon and Nabatieh 5 □ Bekaa

8. Region: 1 □ Urban 2 □ Rural

9. Level of education:
1 □ Secondary 2 □ Technical secondary 3 □ Secondary

4 □ Technical University 5 □ Non-technical University

10. Economic activity:
1 □ Works 2 □ Seeks a job first

3 □ Unemployed 4 □ Studied

5 □ Study and work 6 □ Retired or annuitant

7 □ Other inactive

11. If you work (or have worked), the profession:
0 □ Armed Forces
1 □ Members of the executive and legislative bodies, senior managers of public administration, executives and senior company executives
2 □ Intellectual and scientific professions
3 □ Intermediate professions
4 □ Employees of administrative type
5 □ Service, store and market sellers
6 □ Farmers and skilled workers in agriculture and fisheries
7 □ Artisans and handcraft workers
8 □ Plant and machine operators and assembly workers
9 □ Workers and unskilled workers
10 □ Other specify: ....

Part 3. State of health

12. Are you a smoker?

1 □ Yes 2 □ No

13. If yes, please specify:

Cigarette: a- Number of cigarettes / day: | __ | __ | b- Number of years of smoking: | __ | __ | Nargile: c- Number of nargile / week: | __ | __ | d- Number of years of tobacco: | __ | __ |

14. Are you an alcohol drinker?

1 □ Yes 2 □ No

15. If yes, please specify:

a- 1 □ Occasional or 2 □ Regular

b- Number of glasses / day: | __ | __ |

c- Number of years of consumption: | __ | __ |

16. How do you rate your health?

1 □ Very good 2 □ Good 3 □ Fair 4 □ Bad 5 □ Very bad

17. a- Weight: ׀ ______ ׀ Kg **b-Size:** ׀ __ ׀ __ ׀ __ ׀

18. Do you exercise?

1 □ Yes, low intensity physical activity 2 □ Yes, moderate intensity physical activity

3 □ Yes, high intensity physical activity 4 □ No

19. If yes, how much time do you spend per week? ׀ ____ ׀ hours

Intensity and measurement of physical activity

Physical activity is a behavior that can be characterized by a frequency, intensity, duration and type of practice that define the amount of physical activity in a space-time (day, week, etc.).

MET: The intensity of a physical activity is most often expressed in MET (metabolic equivalent of task), defined as the ratio of energy expenditure related to physical activity on basic metabolism. 1 MET is the level of energy expenditure at rest, sitting on a chair (3.5mL O_2/mn/kg).

Physical activity of low intensity (<3 MET) It does not require effort (no breathlessness, no perspiration)

Examples:

- Iron
- Dust off
- Clean the windows
- To make the beds
- Cook, do the dishes, do the shopping
- Repair and wash the car
- To vacuum
- Sweep gently
- Carry loads up to 6 kg when climbing the stairs
- Clean

Physical activity of moderate intensity (about 3-6 MET)

It requires a moderate effort and significantly accelerates the heart rate. (Moderate shortness of breath, possible conversation, moderate sweating)

Examples:

- walk briskly
- dance
- garden
- engage in traditional hunting and gathering
- actively participate in games and sports with children / take out your pet
- do crafts (For ex: roof repairs, painting)
- lift / move loads <11kg

High intensity physical activity (approximately> 6 MET)

It requires a great effort, the breath is shortened and the heart rate accelerates considerably. (Marked breathlessness, difficult conversation, heavy transpiration)

Examples:

- run
- walk briskly / climb at fast pace
- ride a bike at high speed
- do aerobics
- swim at high speed
- play sports and competitive games (For ex: traditional games, football, volleyball, hockey, basketball)
- doing hard work
- lift / move heavy loads of 11 kg or more

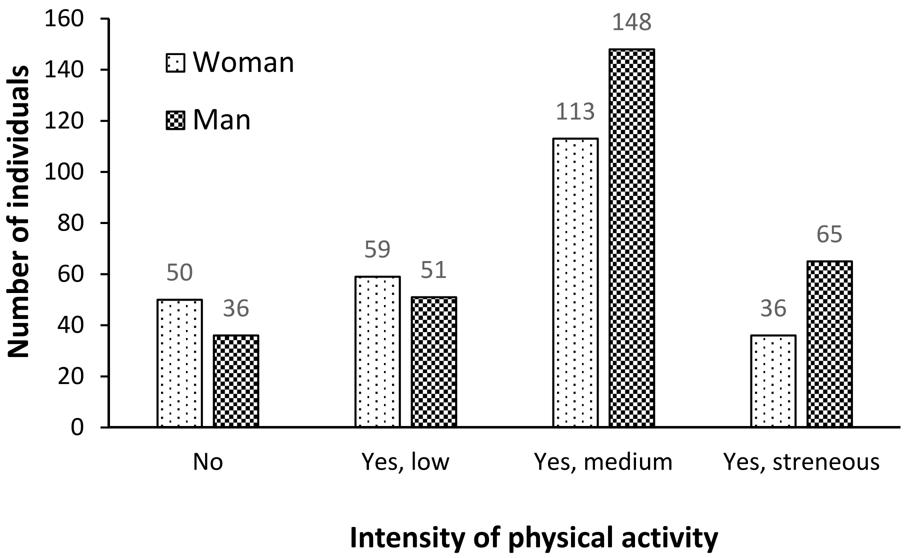

Figure S1: Distribution of physical activity intensity by gender in adult population.

Table S1-a. Cross-tabulation of consumed pita types occurrences for the total 992 respondent in number of individuals.

	White	Brown	Others[1]	Total
White	677			677
Brown	68	162		230
Others[1]	14	16	52	82
White+Brown+Others[1]	3			3
Total	762	178	52	992

[1]Others: rye, 35; Saj, 22; Tannour, 9; whole wheat, 8; oat, 4; bran, 2; baguette, 1; gluten free, 1

Table S1-b. Types of consumed bread for the subpopulation consuming mainly white pita (762 individuals).

Types of consumed bread	Individuals	
	Number	%
White only	677	88.8
White + Brown	68	8.9
White + Others	14	1.8
White + Brown + Others	3	0.4
Total	762	100.0

Table S2: Pearson correlation matrix of the Principal Component Analysis.

Variables	As	Co	Cd	Cr	Hg	Ni	Pb
**As**	1						
**Co**	-0.41*	1					
**Cd**	0.43*	-0.10	1				
**Cr**	-0.03	-0.49*	-0.49*	1			
**Hg**	-0.04	-0.19	-0.53*	0.64*	1		
**Ni**	-0.59*	0.65*	0.09	-0.76*	-0.50*	1	
**Pb**	0.68*	-0.65*	-0.00	0.69*	0.39*	-0.98*	1

* Values significantly different from zero at a level of significance alpha = 0.95

Table S3: **Population categories exposure to Cd, Hg, Cr, Co, Ni, Pb & As trace elements *via* white pita consumption according the most consumed brands.**

Trace element	Brand	Trace element exposure (µg/kg body weigth/day)							
		Children		Teenagers		Women		Men	
		M	95th P	M	95th P	M	95th P	M	95th P
As	B1	0.51[a]	1.86[a]	0.74[a]	2.23[a]	0.50[a]	1.60[a]	0.79[a]	1.61[a]
	B2	1.43[b]	7.65[b]	1.07[ab]	4.17[a]	0.77[b]	2.11[a]	1.05[b]	2.63[a]
	B3	1.54[b]	7.97[b]	1.58[b]	4.48[a]	1.07[c*]	2.19[a*]	1.53[c*]	3.77[b*]
Cr	B1	0.09[a]	0.32[a]	0.13[a]	0.38[ab]	0.09[a]	0.28[a]	0.14[a]	0.28[a]
	B2	0.06[a]	0.30[a]	0.04[b]	0.16[a]	0.03[b*]	0.08[b]	0.04[b*]	0.10[b*]
	B3	1.74[b]	9.00[b]	1.79[c]	5.06[b]	1.21[c*]	2.47[c*]	1.72[c*]	4.26[c*]
Co	B1	0.20[a]	0.72[a]	0.29[ab]	0.86[a]	0.20[ab]	0.31[a]	0.31[a]	0.62[ab]
	B2	0.31[ab]	1.66[a]	0.23[a]	0.91[a]	0.17[a*]	0.23[a*]	0.23[a*]	0.57[a]
	B3	0.41[b]	2.09[a]	0.42[b]	1.18[a]	0.28[b*]	0.40[b*]	0.40[b*]	0.99[b*]
Ni	B1	2.80[a]	10.26[a]	4.06[a]	12.26[a]	2.75[a]	8.83[a]	4.34[a]	8.86[a]
	B2	1.30[a]	6.99[a]	0.97[b]	3.81[a]	0.70[b*]	1.93[b]	0.96[b*]	2.40[b]
	B3	ND	ND	ND	ND	ND	ND	ND	ND
Pb	B1	0.16[a]	0.59[a]	0.23[ab]	0.70[ab]	0.16[a]	0.50[a]	0.25[a]	0.51[a]
	B2	0.72[ab]	3.88[a]	0.54[a]	2.12[a]	0.39[b*]	1.07[b]	0.53[b]	1.33[b]
	B3	1.25[b]	6.43[a]	1.28[b]	3.61[a]	0.86[c*]	1.77[c*]	1.23[c]	3.04[c*]

Trace element	Brand	Trace element exposure (µg/kg body weigth/week)							
		Children		Teenagers		Women		Men	
		M	95th P	M	95th P	M	95th P	M	95th P
Cd	B1	0.10[a]	0.36[a]	0.14[a]	0.43[a]	0.10[a]	0.31[a]	0.15[a]	0.31[a]
	B2	0.22[a]	1.16[a]	0.16[a]	0.64[a]	0.12[a]	0.32[a]	0.16[a]	0.40[a]
	B3	0.19[a]	1.00[a]	0.20[a]	0.56[a]	0.13[a*]	0.28[a*]	0.19[a*]	0.47[a*]
Hg	B1	0.01[a]	0.04[a]	0.02[ab]	0.06[a]	0.01[ab]	0.04[ab]	0.02[a]	0.04[a]
	B2	0.02[ab]	0.09[a]	0.02[a]	0.07[a]	0.01[a*]	0.03[a]	0.01[a*]	0.03[a]
	B3	0.03[a]	0.15[a]	0.03[b]	0.09[a]	0.02[b*]	0.04[b*]	0.03[b*]	0.07[b*]

M = Median, 95th P = 95th percentile. Different letters in each column are significantly different at $P < 0.05$.
*corresponds to a significant difference, respectively, medians and percentiles at $P < 0.05$ for a given brand.
N.D. = Not Determined because the Nickel content was below the detection limit.
